# Immunohistochemical analysis of undifferentiated and poorly-differentiated head and neck malignancies at a tertiary hospital in Nigeria

**DOI:** 10.1186/1758-3284-2-33

**Published:** 2010-11-03

**Authors:** Akinyele O Adisa, Abideen O Oluwasola, Bukola F Adeyemi, Bamidele Kolude, Effiong EU Akang, Jonathan O Lawoyin

**Affiliations:** 1 Department of Oral Pathology, University College Hospital, Ibadan, Oyo State, Nigeria; 2 Department of Pathology, University College Hospital, Ibadan, Oyo State, Nigeria

## Abstract

**Absract:**

This is a retrospective analysis of poorly-differentiated head and neck malignancies at University College Hospital, Ibadan.

Eighty-six poorly-differentiated neoplasms were categorized as carcinomas, sarcomas, lymphomas or neuroendocrine cancers with a panel of 7 antibodies (cytokeratin AE1/AE3, vimentin, desmin, myogenin, leukocyte common antigen and neuron-specific enolase). Immunohistochemical and original hematoxylin-eosin diagnoses were contrasted.

The male: female ratio was 2.5:1, with mean age of 38.9 years. Nasopharynx, nose and maxillofacial bones were the most common locations. Immunohistochemistry confirmed 54.8% of carcinomas, 70.6% of sarcomas and 80% of lymphomas.

Hematoxylin-eosin was able to distinguish between sarcoma and lymphoma but differentiation between a carcinoma and neuroendocrine lesion was poor. Further studies are required to maximize the role of immunohistochemistry as an ancillary diagnostic tool in the West African sub-region.

## Introduction

Histological examination plays a central role in diagnosis, classification, grading and staging of malignancy. Difficulties arise from the subjective nature of histological analysis that are influenced by the practitioner's experience, bias and training. With poorly-differentiated neoplasms, inter- and intra-observer variability can be high [1]. Immunohistochemistry has greatly assisted in the identification of tumors that cannot be accurately identified using routine histopathological procedures [2]. In one study of more than 100 anaplastic tumors, the hematoxylin-eosin diagnosis of carcinoma or lymphoma was revised in approximately 50% of cases following immunohistochemical analysis [3].

In some undifferentiated tumors, subtle features of epithelial versus mesenchymal differentiation can often be appreciated, which assist the immunohistochemical approach to these tumors. Some tumors, however, may not fit into either of these two categories because of their overlapping histological features [4]. Nevertheless, making the correct histopathological diagnosis is essential in deciding the appropriate therapy [5,6].

The immunohistochemical evaluation of undifferentiated tumors should first aim at a broad lineage determination of the neoplasia. Based on the result of the screening panel, a more detailed or specific panel should then be applied to further sub classify the tumor or to confirm a particular diagnosis [4]. The thrust of this study is to evaluate the accuracy of histopathological diagnosis in the broad lineage determination of undifferentiated/poorly-differentiated neoplasms of the head and neck.

## Methodology

1192 head and neck malignancies (oral and nasal cavities, paranasal sinuses, oropharynx, nasopharynx, hypopharynx, larynx, trachea, ear and salivary glands) were retrieved from the archives of the Pathology and Oral Pathology departments of the University College Hospital, Ibadan, Nigeria between 1990 and 2008. 142 poorly-differentiated and undifferentiated neoplasms including anaplastic (undifferentiated) or poorly-differentiated carcinomas, anaplastic large cell lymphomas, pleomorphic sarcomas, malignant fibrous histiocytoma, esthesioneuroblastoma and spindle cell sarcomas were selected. Cases where the original paraffin block could not be obtained were excluded from analysis. Only 86 of the 142 undifferentiated and poorly-differentiated head and neck malignancies diagnosed during the study period satisfied the inclusion criteria.

Freshly prepared sections from each case were stained with hematoxylin-eosin (H&E) and a panel of antibodies to leukocyte common antigen (CD45), cytokeratin AE1/AE3, vimentin, desmin, myogenin and neuron-specific enolase (NSE) using the specifications of the manufacturer (Dako Cytomation, USA).

The sections for immunohistochemistry were de-paraffinized, hydrated and then rinsed in Phosphate Buffered Solution (PBS). They were immersed in heat induced epitope retrieval citrate buffer diluted to 1:10 with distilled water and incubated at 90°C for 1 hour. They were then placed in fresh citrate, cooled in water for 20 minutes and then rinsed in PBS. Positive controls (skin for cytokeratin AE1 or AE3, tonsils for CD45; neural tissue for Neuron-specific enolase; skeletal muscle for Myogenin and Vimentin; and smooth muscle for desmin) and negative controls were employed for each antibody. 3% hydrogen peroxide was added to each section for 10 minutes and the sections were rinsed in 0.1% PBS. The specimens were incubated for an hour with 40-130 μl of appropriately diluted Dako mouse primary antibody, followed by incubation with undiluted labeled polymer Horse Radish Peroxidase conjugated antimouse secondary antibody for 30 minutes. One ml of Diaminobenzidene solution was added to cover the specimen, followed by incubation in a humidity chamber for 15 minutes. The sections were then immersed in aqueous hematoxylin and rinsed in distilled water. The tissue was then dehydrated and subsequently rinsed with xylene. DPX (Distyrene, Plasticizer and Xylene) mounting fluid was then applied and a cover slip placed.

All the seven antibodies used in the panel for one specimen were reviewed sequentially and the pattern and intensity of staining was observed and scored as: negative (0), weakly positive (+1), moderately positive (+2) and strongly positive (+3) [7]. The slides were reviewed without reference to initial histology diagnosis to eliminate bias. The final immunohistochemical findings were then correlated with the H&E stained slides in order to arrive at a final diagnosis.

The data was analyzed using version 16 of the Statistical Package for Social Sciences (SPSS16). Qualitative data were compared using chi-square statistics. Quantitative data were summarized using mean, standard deviation and confidence interval and compared using student t- and/or one-way analysis of variance test. The level of significance was set at p < 0.05. Sensitivity and specificity were calculated using immunohistochemistry as the gold standard to which the original H&E diagnosis was compared. The positive predictive value, negative predictive value, accuracy and degree of agreement were also determined. For degree of agreement, Kappa value >0.75 = excellent agreement, 0.4-0.75 = fair to good agreement and <0.4 = moderate to poor agreement [8].

## Results

The cases comprised 62 (72.1%) males and 24 (27.9%) females. The mean age was 38.9 (SD ± 15.9) with peak occurrence between 25-44 years. The nasopharynx (47.7%), nose (12.8%) and maxillofacial bones (12.8%) were the most common locations.

**Figure 1:**
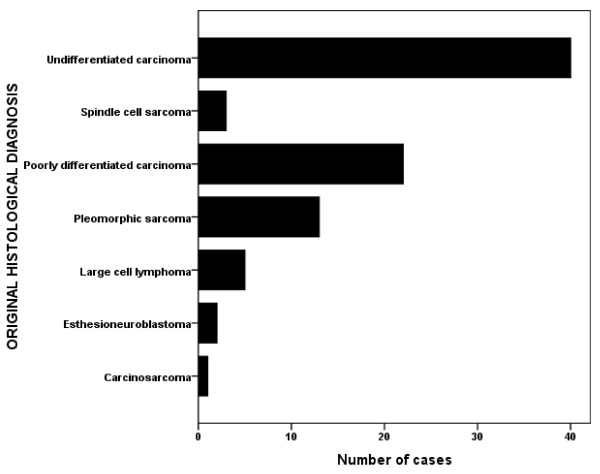
**Histological diagnoses of 86 poorly differentiated/undifferentiated head and neck malignancies**.

The original hematoxylin-eosin diagnoses are shown in figure 1. These diagnoses were confirmed by immunohistochemistry in 34 (54.8%) of the 62 carcinomas, 12 (70.6%) of the 17 sarcomas, four (80%) of the 5 lymphomas and one of the 2 neuroendocrine malignancies (Table 1).

**Table 1:** Comparison of histology diagnosis with immunohistochemical assessment

	IMMUNOHISTOCHEMISTRY DIAGNOSIS						
HISTOLOGY DIAGNOSIS	CA	SARC	LYMPH	NE-CA	CA-SARC	INCONCLUSIVE	TOTAL
**CA**	34	8	8	6	1	5	62
**SARC**	1	12	2	0	0	2	17
**LYMPH**	0	1	4	0	0	0	5
**NE-CA**	0	1	0	1	0	0	2
TOTAL	35	22	14	7	1	7	86

KEY: CA- carcinoma, SARC- sarcoma, LYMPH- lymphoma, NE-CA- neuroendocrine carcinoma.

**Figure 2:**
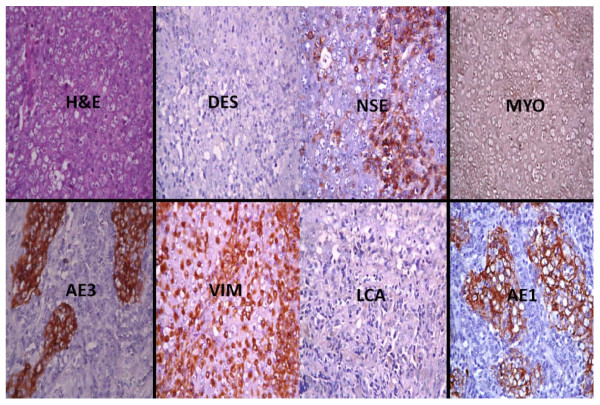
**Photomicrographs showing the Immunohistochemical profile of a neuroendocrine carcinoma**. The haematoxylin and eosin (H&E) section shows highly pleomorphic cells with nuclei vessiculation and prominent nucleoli disposed in islands. Moderate immunopositivity of neurone specific enolase (NSE) for epithelioid-like cells is noted. Epithelial markers (AE1 and AE3) are strongly positive and there is a non-specific staining pattern of epithelial cells and lymphocytes with vimentin (VIM). The tumour is negative for desmin (DES) and leukocyte common antigen (LCA). All immunohistochemical sections were counterstained with hematoxylin. (X400)

**Figure 3:**
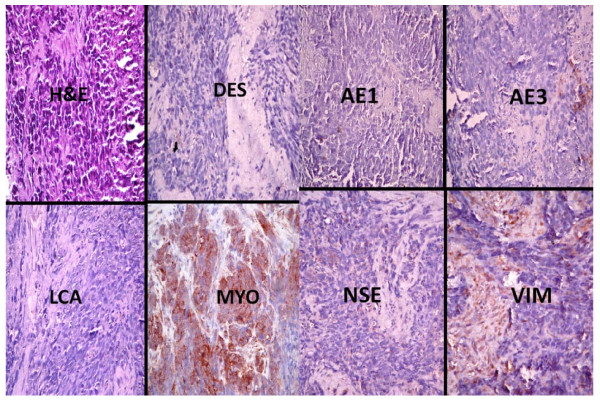
**Photomicrographs showing the immunohistochemical profile of a rhabdomyosarcoma including the haematoxylin and eosin (H&E) slide which displays sheets of pleomorphic and polymorphic cells which have hyperchromatic nuclei**. There is strong immunopositivity for myogenin (MYO), moderate positivity for vimentin (VIM) and equivocal staining with desmin (DES) and neurone specific enolase (NSE). There is also negativity for epithelial markers (AE1 and AE3) and leukocyte common antigen (LCA). All immunohistochemical sections were counterstained with hematoxylin. (X400)

**Figure 4:**
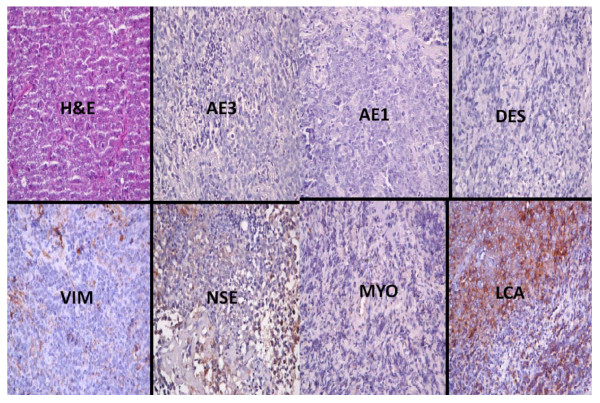
**Photomicrographs show the immunohistochemical profile of a non Hodgkin's lymphoma including the haematoxylin and eosin (H&E) slide that shows a highly cellular lesion with minimal supporting loose connective tissue stroma**. There is strong immunopositivity of the malignant lymphoid cells for leukocyte common antigen (LCA). All of the other immunohistochemical markers (AE1, AE3, myogenin (MYO), vimentin (VIM), desmin (DES), and neuron specific enolase (NSE) are negative. All immunohistochemical sections were counterstained with hematoxylin. (X400).

**Figure 5:**
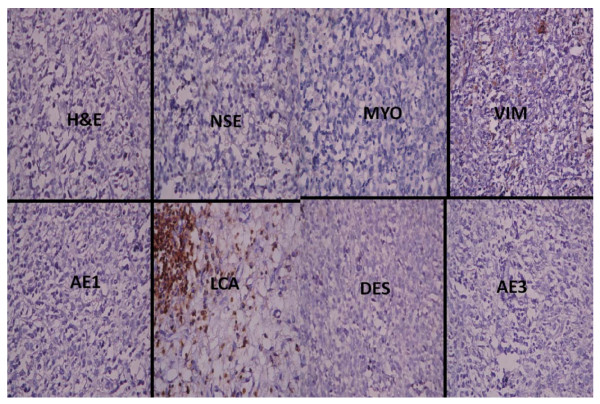
**Photomicrographs are showing the immunohistochemical profile of a lymphoma including the haematoxylin and eosin (H&E) slide which shows some small blue round and spindle cells with indistinct cytoplasm**. There is immunopositivity for leukocyte common antigen (LCA). All of the other immunohistochemical markers are negative. All immunohistochemical sections were counterstained with hematoxylin. (X400).

**Figure 6:**
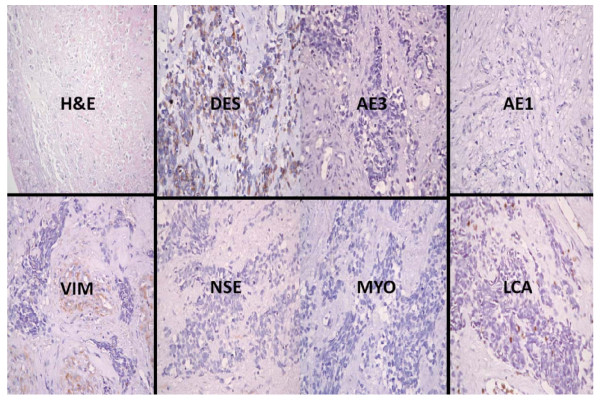
**Photomicrographs show the immunohistochemical profile of a leiomyosarcoma including the haematoxylin and eosin (H&E) slide**. The islands of spindle cells with wavy nuclei show strong immunopositivity for desmin (DES). All of the other markers are negative, with fibrous connective tissue staining for vimentin (VIM). All immunohistochemical sections were counterstained with hematoxylin. (X400).

**Figure 7:**
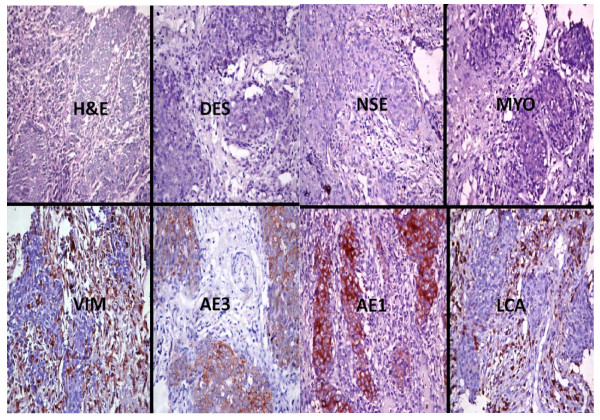
**Photomicrographs show the immunohistochemical profile of a squamous cell carcinoma including the haematoxylin and eosin (H&E) slide which show a follicular arrangement of small round hyperchromatic cells**. There is strong immunopositivity for cytokeratin AE1 and moderate positivity for AE3. All of the other immunohistochemical markers are negative. All immunohistochemical sections were counterstained with hematoxylin. (X400).

Table 2 summarizes the clinical profile of the 33 cases in which there was discordance between the original H&E diagnosis and final diagnosis. Some of these discordant diagnoses are also depicted in figures 2, 3, 4, 5, 6 and 7. Sarcomas, neuroendocrine carcinomas and lymphomas were most often misdiagnosed as carcinomas. There was no obvious correlation between age, gender or site distribution, as compared to diagnosis.

**Table 2:** Age group, gender and topography of head and neck cancers in which the final diagnosis was revised after immunohistochemistry

ORIGINAL HISTOLOGICAL DIAGNOSIS	FINAL DIAGNOSIS AFTER IMMUNOHISTOCHEMISTRY	AGE GROUP (years)	GENDER	TOPOGRAPHY
**Carcinoma**	Neuroendocrine carcinoma	25-44	Male	Lymph node
**Carcinoma**	Neuroendocrine carcinoma	45-64	Female	Nose
**Carcinoma**	Neuroendocrine carcinoma	45-64	Male	Nasopharynx
**Carcinoma**	Neuroendocrine carcinoma	25-44	Female	Nose
**Carcinoma**	Neuroendocrine carcinoma	15-24	Male	Palate
**Carcinoma**	Neuroendocrine carcinoma	≥65	Male	Nasopharynx
**Carcinoma**	Sarcoma	25-44	Male	Face/scalp
**Carcinoma**	Sarcoma	15-24	Male	Nasopharynx
**Carcinoma**	Sarcoma	45-64	Male	Lymph node
**Carcinoma**	Sarcoma	25-44	Male	Nasopharynx
**Carcinoma**	Sarcoma	25-44	Male	Nose
**Carcinoma**	Sarcoma	15-24	Male	Nose
**Carcinoma**	Sarcoma	45-64	Male	Nasopharynx
**Carcinoma**	Sarcoma	45-64	Female	Maxillofacial bone
**Carcinoma**	Lymphoma	≥65	Male	Nasopharynx
**Carcinoma**	Lymphoma	45-64	Male	Nasopharynx
**Carcinoma**	Lymphoma	25-44	Male	Oropharynx
**Carcinoma**	Lymphoma	----	Male	Oropharynx
**Carcinoma**	Lymphoma	15-24	Male	Maxillofacial bone
**Carcinoma**	Lymphoma	45-64	Male	Nose
**Carcinoma**	Lymphoma	25-44	Female	Nasopharynx
**Carcinoma**	Lymphoma	45-64	Female	Nasopharynx
**Carcinoma**	Carcinosarcoma	45-64	Male	Nasopharynx
**Sarcoma**	Carcinoma	45-64	Female	Face/scalp
**Sarcoma**	Lymphoma	15-24	Male	Maxillofacial bone
**Sarcoma**	Lymphoma	25-44	Male	Maxillofacial bone
**Lymphoma**	Sarcoma	25-44	Male	Nose
**Neuroendocrine carcinoma**	Sarcoma	45-64	Male	Nasopharynx

The sensitivity of histology was highest for carcinomas (97.1%) and least for neuroendocrine lesions (14.2%). Specificity of histology was highest for neuroendocrine lesions (98.6%) and lymphomas (98.4%) and least for carcinomas (47.7%). The positive predictive values of histology was highest for sarcomas and lymphomas (80% each), while the negative predictive value was highest for carcinomas (95.4%). Accuracy of histology was highest for the neuroendocrine tumors (91.1%). The level of agreement between histology and immunohistochemistry, given by the kappa values, was highest for sarcomas (54%) (Table 3).

**Table 3:** Sensitivity and specificity of histology

	Carcinomas	Sarcomas	Lymphomas	Neuroendocrine carcinomas
**Sensitivity**	0.971	0.545	0.285	0.142
**Specificity**	0.477	0.947	0.984	0.986
**PPV**	0.596	0.800	0.800	0.500
**NPV**	0.954	0.843	0.864	0.922
**Accuracy**	0.696	0.835	0.860	0.911
**Kappa statistics value**	0.42	0.54	0.42	0.18

PPV - positive predictive value, NPV - negative predictive value

## Discussion

In the present study, the original histological diagnosis of 62 lesions was carcinoma, but only 34 (54.8%) were confirmed by immunohistochemistry. This proportion is higher than the 27.9% confirmation rate of carcinomas in a study by Bianchini *et al *in Italy [9]. Lesions that were confused with carcinomas included 8 (12.9%) sarcomas, 8 (12.9%) lymphomas, 6 (9.7%) neuroendocrine tumors and 1 carcinosarcoma. Gatter [10] also reported in a study that 29 cases (67.4%) from 43 cases initially thought to be anaplastic carcinomas were revised as lymphomas by immunohistochemistry. This demonstrates the value of immunohistochemistry in distinguishing between anaplastic carcinomas and malignant lymphomas of the head and neck, as the latter is much more amenable to treatment than the former. This study thus corroborates other studies which suggest that immunohistochemical technique has a role in the definition of undifferentiated tumors.

Eighty percent of lymphomas diagnosed by histology in this study were confirmed by immunohistochemistry and this is comparable to the 66% reported confirmation of lymphomas in another study [3].

After the immunohistochemical analysis, almost 10% of the lesions thought to be undifferentiated carcinomas were revised to neuroendocrine carcinomas. More than 60% of these revised lesions were found either in the nose or nasopharynx and occurred more commonly in males (66.7%). Histopathological differentiation of undifferentiated carcinoma from neuroendocrine carcinoma is challenging and is significantly aided by immunohistochemistry [11].

The present study had inconclusive diagnosis by immunohistochemistry in 8.1% of cases, Bianchini *et al *reported 18.6% and Gatter reported 6.7% inconclusive results. This could be due to technique differences, different antigen retrieval methods or the absence of the antigen suspected. Use of inappropriate antibodies may also be responsible for absence of immunoreactivity. In addition, some poorly-differentiated tumors might require other techniques such as electron microscopy and molecular studies before an accurate diagnosis can be achieved [12,13].

In this study, histology, to a reasonable extent is able to determine if a sarcoma or lymphoma is present or if they are absent. However it cannot, with a good degree of certainty, determine if a carcinoma or a neuroendocrine tumor is present, although it can exclude them fairly accurately. The high number of carcinomas seen in this study therefore suggests an over-diagnosis of carcinomas by histology. For correct management to be instituted for any malignant lesion it must be diagnosed accurately. In this study the diagnostic accuracy of histology for carcinomas is 69.6%. This means that almost 70% of the time, histology will diagnose carcinomas accurately. The diagnostic accuracy for sarcomas, lymphomas and neuroendocrine tumors is 83.5%, 86% and 91.1% respectively. Therefore further ancillary tests will be needed to resolve diagnostic doubts.

The level of agreement in this study between morphological classification by histology and immunohistochemical assessment was fair for sarcomas (kappa = 0.54), moderate for carcinomas and lymphomas (kappa = 0.42), and poor for neuroendocrine tumors (kappa = 0.18). However, a larger sample of neuroendocrine tumors will be required before any affirmative deductions can be made about them.

Undifferentiated carcinomas were the most prevalent group in this study constituting 64% of the undifferentiated malignancies and 7.6% of all head and neck malignancies. A study by Gatter *et al *[10] in the United Kingdom recorded poorly-differentiated lymphomas (44.2%) as the most prevalent undifferentiated tumors. Bianchini *et al *[9] however reported in their study that the most prevalent cell pattern for poorly-differentiated tumors was the round cell pattern (51%). They only grouped these lesions into their histogenetic lineage after immunohistochemistry and not before [9]. In practice however a protocol should be developed, guiding the choice of panel of antibodies based on cellular morphology to reduce 'wastage' of antibodies.

## Conclusion

In this study, test of sensitivity and specificity show that histology is able to determine the presence or absence of a sarcoma or lymphoma to a good extent but confirmation of a carcinoma or neuroendocrine lesion is poor, although it is able to exclude them fairly accurately. This study has also shown that the use of antibodies in immunohistochemistry greatly assist in the identification of tumors which cannot be accurately identified using routine histopathological procedures.

More detailed studies for specific pathological conditions need to be carried out in order to maximize immunohistochemistry as an ancillary diagnostic tool and expand its versatility in Nigeria and the West African sub-region.

## Conflict of interest statement

The authors declare that they have no competing interests.

## Authors' contributions

AO, AO and JO conceived of the study, and participated in its design and coordination and helped to draft the manuscript. EEU, BF and B participated in the design of the study, drafting of the manuscript and performed the statistical analysis. AO, AO and EEU participated in the revision and reporting of the cases. All authors read and approved the final manuscript.

## References

[B1] Taylor CR An exaltation of experts: concerted efforts in the standardization of immunohistochemistry Appl Immunohistochem 1993 1 232 243 10.1016/0046-8177(94)90164-3 7508882

[B2] Ramaekers F Spandidos D Current perspectives on molecular and cellular oncology 1992 JAI Press: London 285 318

[B3] Gatter KC Alcock C Heryet A Mason DY Clinical importance of analyzing malignant tumors of uncertain origin with immunohistological techniques Lancet 1985 1 8441 1302 5 10.1016/S0140-6736(85)92794-1 2860495

[B4] Bahrami A Truong LD Ro JY Undifferentiated Tumor: True Identity by Immunohistochemistry Arch Pathol Lab Med 2008 132 3 326 348 18318577 10.5858/2008-132-326-UTTIBI

[B5] Michaels L Michaels L Neuroectodermal tumors Ear, Nose and Throat Histopathology 1987 Berlin: Springer-Verlag 89

[B6] Triche TJ Diagnosis of small round cell tumors of childhood Bull Cancer 1988 75 3 297 310 3285915

[B7] IHC World http://www.ihcworld.com/ihc_scoring.htm Accessed on the 7/7/09

[B8] Kirkland BR Sterne JA Essential of Medical Statistics 2 434

[B9] Bianchini WA Altemani AM Paschoal JR Undifferentiated head and neck tumors: the contribution of immunohistochemical technique to differential diagnosis Sao Paulo Med J 2003 121 6 10.1590/S1516-31802003000600005 PMC11110635 14989140

[B10] Gatter KC Alcock C Heryet A Pulford KA Heyderman E Taylor-Papadimitriou J Stein H Mason DY The differential diagnosis of routinely processed anaplastic tumors using monoclonal antibodies Am J Clin Pathol 1984 82 1 33 43 6741874 10.1093/ajcp/82.1.33

[B11] Harrison LB Sessions RB Hong WK Head and Neck Cancer. Google Books page 459. Accessed on 28/July/2009

[B12] Dar AU Hird PM Wagner BE Underwood JC Relative usefulness of electron microscopy and immunocytochemistry in tumor diagnosis: 10 years of retrospective analysis J Clin Pathol 1992 45 8 693 696 10.1136/jcp.45.8.693 1401179 PMC495146

[B13] Dolan M Snover D Comparison of immunohistochemical and fluorescence in situ hybridization assessment of HER-2 status in routine practice Am J Clin Pathol 2005 123 5 766 770 10.1309/Q0DGL26RUCK1K5EV 15981817

